# Neural characteristics of cognitive reappraisal success and failure: An ERP study

**DOI:** 10.1002/brb3.1584

**Published:** 2020-03-11

**Authors:** Dan Cao, Yingjie Li, Margaret A. Niznikiewicz

**Affiliations:** ^1^ Shanghai Institute for Advanced Communication and Data Science School of Communication and Information Engineering Qianweichang College Shanghai University Shanghai China; ^2^ Laboratory of Cognitive Neuroscience Boston VA Healthcare System Brockton Division and Department of Psychiatry Harvard Medical School Boston MA USA

**Keywords:** increased late positive potentials, P200 component, reappraisal success and failure

## Abstract

**Introduction:**

Cognitive reappraisal, an important strategy of emotion regulation, can change emotional experience and attention to emotional information. However, not all individuals can deploy reappraisal strategies successfully. In the current study, we investigated event‐related potential (ERP) characteristics of reappraisal success and of reappraisal failure.

**Methods:**

Twenty‐six participants were divided into the success group or the failure group based on self‐report ratings of how successful they were in reducing their response to negative images using cognitive reappraisal strategy. All participants viewed 30 neutral images and 30 negative images which they were asked to just watch, and 30 negative stimuli that they were asked to reappraise, while electroencephalogram (EEG) was recorded.

**Results:**

The success group reported a significant reduction in the unpleasantness of negative images than the failure group in the negative‐reappraisal condition. The ERP data indicated that two time windows differentiated between the success and failure groups. In 200–300 ms, P200 was significantly more positive to the negative‐watch condition relative to both negative‐reappraisal and neutral conditions in the failure group, while no difference was observed in the success group. In 300–5,000 ms, cognitive reappraisal led to increased late positive potential (LPP) relative to negative‐watch in the early and middle latency windows (300–3,100 ms) in both groups; in the late latency window (3,100–5,000 ms), the reappraisal success group showed the LPP amplitude to the negative‐reappraisal stimuli to be more positive than to the negative‐watch stimuli, while no difference was found in the reappraisal failure group.

**Conclusion:**

Our study provided direct evidence that different neurophysiological features were associated with reappraisal success and failure while engaging in the reappraisal of negative stimuli. This result will contribute to better understanding of the neural mechanism of emotion regulation in emotional disorders (i.e., depression and anxiety).

## Graphical abstract

Our work analyzed behavioral and neurophysiological (event‐related potentials [ERPs]) indices according to whether the participants succeeded (success group) or not (failure group) in reducing negative reaction to negative stimuli based on behavioral responses. More positive P200 was observed to negative‐watch stimuli relative to both negative‐reappraisal and neutral stimuli in the failure group in the occipital region. And cognitive reappraisal increased late positive potential (LPP) amplitude relative to negative‐watch in the LPP early (300–1,700 ms) and middle (1,700–3,100 ms) latency windows in both reappraisal success and failure groups. At LPP late latency window (3,100–5,000 ms), reappraisal success group showed the difference between the negative‐watch and negative‐reappraisal conditions, while no difference was found in the failure group.

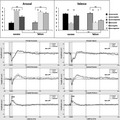

## 1 INTRODUCTION

Emotion regulation refers to the ability to modify the intensity of emotional experience and attention to emotional information (Gross & Thompson, 2007). Cognitive reappraisal, one of the most important cognitive strategies of emotion regulation, aims to reinterpret the meaning of an emotional event or stimulus (Buhle et al., 2014; Foti & Hajcak, 2008; Gross & John, 2003; Hajcak & Nieuwenhuis, 2006; Ochsner & Gross, 2005). Reappraisal appears to be highly effective in down‐regulating the experience of negative emotions with few cognitive and physiological costs (Gross, 1998, 2002; Hermann, Kress, & Stark, 2017; Ochsner, Silvers, & Buhle, 2012; Shafir, Schwartz, Blechert, & Sheppes, 2015; Silvers, Buhle, Ochsner, & Silvers, 2013). The effects of reappraisal aimed to decrease negative emotions are reflected behaviorally in the reduction of *self‐reported* negative experience (Staudinger, Erk, Abler, & Walter, 2009; Wager, Davidson, Hughes, Lindquist, & Ochsner, 2008), that is, reduced unpleasantness of, and arousal associated with, negative stimuli (Foti & Hajcak, 2008; Hajcak & Nieuwenhuis, 2006; Thiruchselvam, Blechert, Sheppes, Rydstrom, & Gross, 2011; Van Cauwenberge, Leeuwen, Hoppenbrouwers, & Wiersema, 2017; Yuan, Zhou, & Hu, 2014). Furthermore, numerous studies suggest that reappraisal can influence many aspects of emotional responding, such as self‐reported negative affect (Gross, 1998), peripheral physiology (Ray, McRae, Ochsner, & Gross, 2010), and neural indicators of emotional arousal (Hajcak & Nieuwenhuis, 2006). However, not all individuals can deploy reappraisal strategies successfully.

In behavioral studies, reappraisal success has been defined as the decrease in unpleasantness of negative stimuli in the self‐reported emotion assessment and the lack of such a decrease has been defined as reappraisal failure (Wager et al., 2008). Brain functional studies, using either functional magnetic resonance imaging (fMRI) or event‐related potential (ERP) methodologies, have focused on neural correlates of reappraisal processes. Both types of studies examined either neural or behavioral correlates of the reappraisal success or of the reappraisal failure. However, to the best of our knowledge, there are no published studies that focused on assessing both the success and the failure of reappraisal at the same time.

Among the studies that focused on the reappraisal success, Wager et al. (2008), using fMRI, tested how the activity of prefrontal cortex was related to reappraisal success: Reappraisal‐induced activation in the right ventrolateral prefrontal region was found to be positively related to the activation in the nucleus accumbens and was negatively related to the activation of the ventral amygdala. Staudinger et al. (2009) found that reappraisal success was associated with a trend toward positive correlation with the ventral striatum activation. Shiota and Levenson (2009) using standardized emotional stimuli and a multimethod approach (subjective experience, physiological response, and subjects' facial expression) assessed the relationship between age and different emotion regulation strategies associated with regulation success. The results indicated that younger participants were more successful when they used a detached reappraisal strategy, while older adults felt more successful when they used a positive reappraisal strategy. McRae, Ciesielski, and Gross (2012) further explored the question of different strategies and different goals that individuals might adopt while engaging in reappraisal and pointed out that reappraisal could be implemented in the service of different emotional goals (i.e., increased positive affect and decreased negative affect) by using different strategies.

A few studies (e.g., Gardener, Carr, MacGregor, & Felmingham, 2013; Sarlo, Übel, Leutgeb, & Schienle, 2013) reported on reappraisal failures showing that unsuccessful reappraisal of negative images did not change the emotional intensity based on self‐report.

In the current study, we have adopted an event‐related potential (ERP) methodology with its excellent temporal resolution to track the unfolding of neural events to address the question of neural signatures of the reappraisal success and of reappraisal failure. ERPs have been previously successfully applied to investigate neural mechanisms underlying cognitive reappraisal. For example, Hajcak and Nieuwenhuis (2006) focused on the impact of reappraisal on the amplitudes of ERP components underlying the reappraisal process.

The effects of reappraisal on the ERP components have varied across studies. In some studies, the ERP effects associated with reappraisal have been observed at earlier latencies, for example, at ~200 ms in Hajcak and Nieuwenhuis study (2006) or at ~400 ms in Moser et al. study (2009) and in Foti and Hajcak study (2008). Other studies reported on ERP effects observed at longer latencies, after 1,500 ms, after the stimulus onset (Parvaz, MacNamara, Goldstein, & Hajcak, 2012; Thiruchselvam et al., 2011).

The ERP component most often reported as an index of neurocognitive changes associated with the reappraisal has been the late positive potential (LPP), a positive‐going component starting approximately 300 ms after the stimulus onset and maximal at central–parietal sites. In addition to reappraisal, in several studies, the LPP has been demonstrated to be sensitive to emotional stimuli with larger amplitudes observed to emotional relative to neutral stimuli (Foti & Hajcak, 2008; Hajcak & Olvet, 2008; Parvaz et al., 2012; Schupp et al., 2000; Schupp, Junghöfer, Weike, & Hamm, 2003).

A number of studies reported that the LPP amplitude changed in the course of reappraisal process. In several of these studies, reappraisal was associated with the reduction of LPP amplitude when negative stimuli were reappraised successfully, that is, interpreted as neutral (Foti & Hajcak, 2008; Hajcak & Nieuwenhuis, 2006; Parvaz et al., 2012; Thiruchselvam et al., 2011). In contrast, more recent studies, in which reappraisal processes were initiated at the onset of the negative stimulus, reported an increased LPP in the reappraisal condition (e.g., Langeslag & Surti, 2017) likely reflecting more effort associated with engaging in reappraisal. Finally, some studies did not show the effect of reappraisal on the LPP amplitude. For example, Gardener et al. (2013) found no changes in the LPP indexing the reduction in negative emotions compared to the maintain condition. Yuan et al. (2014) also failed to find the effect of reappraisal on the LPP amplitude.

These contradictory results seem to indicate that reappraisal success and reappraisal failure may lead to different neurocognitive changes as indexed by LPP. Thus, careful investigation of conditions leading to both the success and the failure in changing emotional response to a negative emotion is necessary. In this study, we addressed the question of how the brain activity related to the reappraisal failure is different from the activity to the reappraisal success using ERP evidence.

## 2 MATERIALS AND METHODS

### 2.1 Participants

Twenty‐six right‐handed, healthy participants participated in the experiment. All participants were recruited from Shanghai University. Based on self‐report, they had no history of neurological or psychiatric illness; none of them used psychoactive medication; they had no history of substance or alcohol abuse. Each participant had normal or corrected‐to‐normal vision. All participants filled out self‐rating anxiety scale (SAS) and self‐rating depression scale (SDS). Based on their scores, they were not depressed or anxious. All participants signed an informed consent form before the experiment and were paid for participation. The experimental protocol was approved by Shanghai Ethics Committee for Clinical Research. The study was conducted in accordance with the Declaration of Helsinki.

### 2.2 Materials

Ninety color images^1^The codes of the IAPS images used are as follows: Negative—1111, 2141, 2750, 3350, 6315, 6560, 9001, 9120, 9410, 9428, 1050, 2276, 2745.2, 3530, 6313, 6571, 9000, 9181, 9342, 9433, 1525, 2683, 2900.1, 6211, 6370, 6831, 9040, 9252, 9421, 9600, 1300, 2399, 2751, 6200, 6350, 6830, 9006, 9250, 9420, 9571, 1930, 2375.1, 3181, 3550, 6415, 6821, 9041, 9254, 9423, 9570, 2095, 2691, 3220, 6230, 6510, 7359, 9042, 9265, 9424, 9635.1; Neutral—2190, 2440, 2579, 5510, 7205, 2191, 2480, 2580, 6150, 7242, 2235, 2493, 2749, 7052, 7550, 2272, 2495, 2840, 7090, 7950, 2280, 2518, 2850, 7140, 9210, 2393, 2570, 5471, 7009, 7190. (60 negative, 30 neutral), size 260 × 195 pixels, were chosen from the International Affective Picture System (IAPS; Lang, Bradley, & Cuthbert, 1999). The images were controlled for arousal and valence. Valence scale spanned from 1 to 9 with 1 = most negative and 9 = most positive. Arousal scale spanned from 1 to 9 as well, with 1 = calm and 9 = aroused. The two image categories differed in valence (*M* = 5.10, *SD* = 0.38 for neutral; *M* = 2.55, *SD* = 0.52 for negative) and arousal (*M* = 3.14, *SD* = 0.47 for neutral; *M* = 5.75, *SD* = 0.81 for negative). The 30 neutral images were used in the neutral‐view condition where subjects were asked to just view the pictures. The 60 negative images were randomly divided into two sets of 30 images, equated for both valence and arousal (all *p*‐values > .1). One set was used in the negative‐watch condition where participants were asked to just view the pictures, and another set was used in the negative‐reappraisal condition where the participants were asked to engage in the reappraisal strategies as soon as they saw a negative valance picture.

The images were presented on a color monitor using E‐prime 2.0 stimulus presentation software (Psychology Software Tools), at a viewing distance of approximately 70 cm, on an LCD screen (17‐inch), with each image presented at an approximately 40° of visual angle horizontally and vertically. All testing was conducted in a sound‐attenuated EEG chamber.

### 2.3 Procedure

We used the paradigm described in detail in Thiruchselvam et al. (2011). The experimenter explained the cognitive strategy of cognitive reappraisal to every participant at the beginning of the experiment. The participants were told that cognitive reappraisal aims to change the emotional response to negative images. Participants were instructed to decrease the intensity of the emotion they felt in response to the picture. For example, they could imagine the content of the images as if from a movie clip, or to imagine the content of the images as having a positive outcome (Gardener et al., 2013; Langeslag & Surti, 2017). They were then guided through several practice trials. During the practice trials, participants were instructed that they should begin implementing the strategy of cognitive reappraisal only after the image appeared on the screen. After each practice, trial participants were asked to report how they altered their emotional approach to the image and whether they felt more neutral in response to a negative image. Following the training, EEG sensors were attached and participants were engaged in either viewing the images or performing an emotion regulation task.

#### 2.3.1 The emotion regulation task

The emotion regulation task consisted of three blocks of 30 trials each. For all trials, participants were required to keep their eyes on the screen. Each block included three types of tasks with 10 trials each: VIEW (neutral image), WATCH (negative image), and REAPPRAISAL (negative image). For both the VIEW and WATCH trials, participants were instructed to simply look at the presented image. The VIEW trials were considered the baseline. The WATCH trials were intended to elicit an unregulated form of emotional responding to negative images. The REAPPRAISAL trials aimed to capture neurocognitive processes associated with efforts to reduce the degree of negative emotion experienced by the participant.

**Figure 1:**
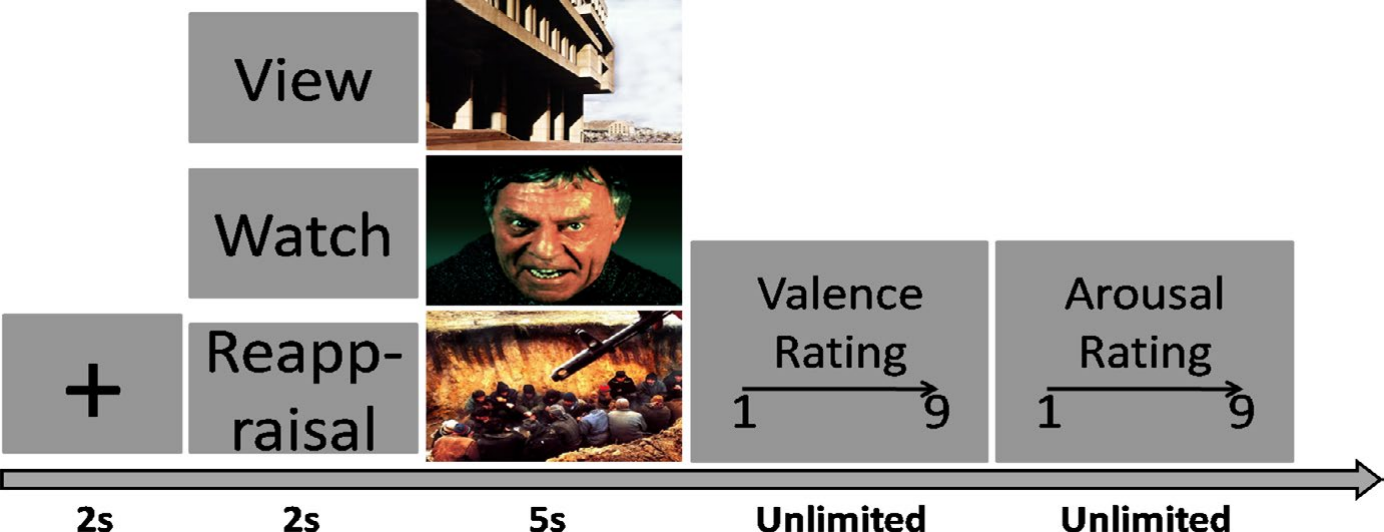
Trial structure for the regulation task. A black fixation cross appeared in the center of a gray screen for 2 s, followed by an instruction cue (either VIEW, WATCH, or REAPPRAISAL) for 2 s. An image was then displayed for 5 s. After the offset of each image, the self‐report ratings on the dimensions of valence and arousal were presented separately, with the range of the rating scale of 1–9. For valence, the rating of 1 stood for “highly negative” and “9” stood for “highly positive”; for arousal, the rating of “1” stood for “completely calm” and “9” stood for “highly exciting”

The structure of the regulation task is illustrated in Figure 1. A black fixation cross appeared in the center of a gray screen for 2 s, followed by an instruction cue (either VIEW, WATCH, or REAPPRAISAL) for 2 s. An image was then displayed for 5 s against a gray background. After the offset of each image, participants rated the image on the dimensions of valence and arousal separately, with the range of the rating scale of 1–9. For valence, the rating of 1 stood for “highly negative” and “9” stood for “highly positive”; for arousal, the rating of “1” stood for “completely calm” and “9” stood for “highly exciting.” The next trial began after participants completed the ratings. The sequence of the 30 trials within each block was randomized for each participant, and the order of the blocks was counterbalanced. Participants took a break of about 1 min between the blocks.

#### 2.3.2 Behavioral criteria for the reappraisal success and failure groups

Reappraisal success was defined as the decrease in the ratings of emotional experience (i.e., valence and arousal) when reappraisal was applied to negative images relative to when the negative images were watched only (Shiota & Levenson, 2009; Wager et al., 2008). When the decrease was not observed, it was considered a reappraisal failure. Accordingly, for each participant, we conducted paired *t* tests of valence and arousal ratings between 30 negative‐watch trails and 30 negative‐reappraisal trials. If the valence ratings for negative‐reappraisal trials were significantly higher than valence ratings for negative‐watch trials (*p* < .05), and the arousal ratings for negative‐reappraisal trials were significantly lower than arousal ratings for negative‐watch trials (*p* < .05), reappraisal was considered a success and the participant was assigned to the success group. Otherwise, the participant was assigned to the failure group. Based on their scores, 13 participants were included in the success group (male/female = 7/6; mean age = 22.84 ± 0.80; mean year of education = 16.69 ± 0.48) and 13 participants were included in the failure group (male/female = 7/6; mean age = 23.00 ± 0.71; mean years of education = 16.77 ± 0.60). No statistical difference (*t* test, *p* > .05) was found in both groups in terms of demographic characteristics including age and education and of the SAS and SDS scores (see Table 1).

**Table 1:** Demographic and affective characteristics of subjects in the success and failure groups (mean ± *SD*)

	Success	Failure	*p*
Cases (*n*)	13	13	
Handedness (left/right)	0/13	0/13	
Age (years)	22.84 ± 0.80	23.00 ± 0.71	.608
Education (years)	16.69 ± 0.48	16.77 ± 0.60	.721
SAS score	35.62 ± 4.93	34.73 ± 5.56	.685
SDS score	39.31 ± 9.37	38.46 ± 4.88	.795
Gender			
Male	7	7	
Female	6	6	

### 2.4 EEG recording and data processing

While participants performed the emotion regulation task, EEG was recorded using SynAmps amplifiers and digitized with Scan 4.3 software (Neuroscan, Inc.). EEG recordings were obtained with standard Ag/AgCl 32‐channel scalp electrodes, the 10–20 system, FP1/2, F3/4, F7/8, FC3/4, FT7/8, C3/4, T3/4, CP3/4, TP7/8, P3/4, T5/6, O1/2, Fz, FCz, Cz, CPz, Pz, Oz. EEG was referenced to the right mastoid; an AFz ground was used. Electrooculogram (EOG) electrodes were positioned above and below the left eye as well as on the outer canthi of each eye. EEG was continuously recorded at a sampling frequency of 1,000 Hz and band‐pass filtered from 0.05 to 100 Hz, and interelectrode impedance was kept below 10 kΩ.

Data preprocessing was completed with EEGLAB (version 12.0.2.6b) and ERPLAB (version 6.1.3) MATLAB packages. Data were re‐referenced to the average of the left and right mastoids, and low‐pass filtered at 13 Hz. EEG was inspected visually for the presence of artifacts. Then, eyeblinks and muscle artifacts were removed using an independent component analysis approach (Delorme & Makeig, 2004). Single‐trial EEG epochs were extracted with a 500 ms prestimulus baseline, time locked to the stimulus onset, and spanning the entire duration of the image presentation (5,000 ms). EEG epochs with artifacts (>±100 μV) were excluded. All participants' data used for final analysis had at least 21 “good” trials for each condition. The number of “good” trials did not differ significantly by condition and group. Artifact‐free ERPs were constructed by separately averaging trials in the three conditions: neutral‐view, negative‐watch, and negative‐reappraisal. The resulting ERPs were baseline‐corrected, using the entire 500 ms baseline.

### 2.5 ERP analysis

#### 2.5.1 N100 and P200

The N100 was measured as the peak amplitude between 100 and 200 ms, and the P200 was measured as the peak amplitude between 200 and 300 ms, poststimulus, in the occipital region (O1, Oz, O2).

#### 2.5.2 LPP

Late positive potential was measured in the frontal (FP1, FP2, F3, F4), the central (Cz and CPz), and the occipital (O1, O2, Oz) regions. Based on the grand averages, we scored the early stage of the LPP from 300 to 1,700 ms poststimulus onset since the LPP was considered to start approximately 300 ms after the stimulus onset (Hajcak, Dunning, & Foti, 2009; Thiruchselvam et al., 2011) and to reach a maximum at 1,688 ms (MacNamara, Foti, & Hajcak, 2009). Then, we visually inspected the grand average waveforms of ERP for both groups (see Figure 3) and found that there was an inflection point approximately at 3,100 ms in the LPP component, especially in the central site. Thus, we selected 1,700–3,100 ms as the middle stage and 3,100 ms to the end of the epoch duration (5,000 ms) as the late stage of the LPP.

### 2.6 Statistical analysis

#### 2.6.1 Behavioral data

After the two groups were identified based on paired *t* tests as described above, valence and arousal as a function of experimental stimulus type were examined for the success group and for the failure group. We used repeated‐measures analysis of variance (ANOVA) with one within‐group factor of Condition (neutral, negative, and reappraisal) and one between‐group factor of Group (success and failure) for valence and arousal, separately, assessed after the experimental exposure to the stimuli.

#### 2.6.2 ERP

##### N100 and P200

Repeated‐measures ANOVAs were used to examine N100 and P200 amplitudes in the occipital region, with one within factors of Condition (neutral, negative‐watch, and negative‐reappraisal) and one between‐group factor of Group (success and failure).

##### LPP

Repeated‐measures ANOVAs were performed with the two within factors of Condition (neutral, negative‐watch, and negative‐reappraisal) and Area (frontal, central, and occipital) and one between factor of Group (success and failure), for the early (300–1,700 ms), middle (1,700–3,100 ms), and late (3,100–5,000 ms), separately. The simple‐effects analysis was performed if any interaction between factors was found. All analyses were conducted at the .05 level of significance. Multiple comparisons were corrected with Bonferroni correction.

Pearson's correlations were conducted in both groups between the LPP amplitude and the behavioral results, that is, for the valence difference and the arousal difference between the negative‐watch and the negative‐reappraisal conditions, separately.

All statistical analyses were conducted using SPSS 22.0.

## 3 RESULTS

### 3.1 Behavioral data results

**Figure 2:**
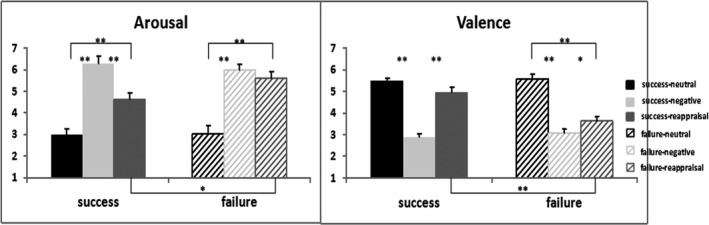
Behavioral results of arousal and valence ratings in the success and in the failure groups. For arousal, in the success group, both negative‐watch and negative‐reappraisal elicited greater arousal than the neutral stimuli; negative‐reappraisal led to greater reduction of arousal than negative‐watch; in the failure group, both negative‐watch and negative‐reappraisal elicited greater arousal than the neutral stimuli, while negative‐reappraisal did not reduce the arousal relative to negative‐watch. In addition, negative‐reappraisal significantly reduced more arousal in the success group than in the failure group. For valence, in the success group, the negative‐watch condition was associated with greater unpleasantness than the neutral stimuli and the negative‐reappraisal conditions. The valence of negative‐reappraisal stimuli was similar to the neutral stimuli; in the failure group, negative‐watch stimuli elicited greater unpleasantness than the neutral stimuli and the negative‐reappraisal stimuli and negative‐reappraisal stimuli elicited more unpleasantness than the neutral stimuli. In addition, negative‐reappraisal reduced more unpleasantness in the success group than the failure group. **p < .01 and *p < .05

Table 2 and Figure 2 show the ratings of arousal and valence of the neutral, negative‐watch, and negative‐reappraisal stimuli for the success and failure groups.

**Table 2:** Mean valence and arousal ratings (*SD* in parentheses) of each condition for the success and failure groups

Condition	Success		Failure	
	Arousal	Valence	Arousal	Valence
Neutral	2.97 (0.29)	5.49 (0.16)	3.05 (0.40)	5.60 (0.23)
Negative‐watch	6.33 (0.31)	2.89 (0.15)	5.99 (0.30)	3.09 (0.18)
Negative‐reappraisal	4.63 (0.33)	4.97 (0.24)	5.62 (0.31)	3.64 (0.25)

Note

1 = negative/calm, 9 = positive/aroused.

For arousal, the main effect of Condition (*F*(2,48) = 95.49, *p* < .001, *η* ^2^ = .799) revealed that negative‐watch elicited greatest arousal and the neutral stimuli were associated with lowest arousal (*p* < .001). The main effect of Group was not significant (*p *= .526).

There was also the interaction effect of Condition*Group (*F*(2,48) = 4.304, *p* = .019, *η* ^2^ = .152). To follow up on the Condition*Group interaction, we conducted further analyses:

1. The ANOVA with a within factor of Condition was performed in each group. In the success group, the main effect of Condition (*F*(2,24) = 55.755, *p* < .001, *η* ^2^ = .823) revealed that both negative‐watch and negative‐reappraisal elicited higher arousal than the neutral stimuli (*p* < .001, respectively); and negative‐reappraisal was associated with lower arousal than negative‐watch (*p* = .001). In the failure group, the main effect of Condition (*F*(2,24) = 44.746, *p* < .001, *η* ^2^ = .789) revealed that both negative‐watch and negative‐reappraisal elicited greater arousal than the neutral stimuli (*p* < .001, respectively), while negative‐reappraisal did not reduce the arousal relative to the negative‐watch condition (*p* = .379).
2. The independent *t* tests of between‐group comparisons were performed for each condition. This analysis showed that negative‐reappraisal significantly reduced more arousal in the success group than in the failure group (*t* = −2.174, *p* = .040).

For valence, the main effect of Condition (*F*(2,48) = 91.455, *p* < .001, *η* ^2^ = .792) revealed that the ratings to the negative‐watch stimuli were lowest and to the neutral stimuli were highest (*p* < .001). The main effect of Group was not significant (*p* = .084).

There was also the interaction effect of Condition*Group (*F*(2,48) = 10.309, *p* < .001, *η* ^2^ = .300). To follow up on the interaction of Condition*Group, we conducted further analyses:

1. The ANOVA with a within factor of Condition was performed in each group. In the success group, the main effect of Condition (*F*(2,24) = 63.012, *p* < .001, *η* ^2^ = .840) revealed that negative‐watch elicited greater unpleasantness than the neutral stimuli (*p* < .001) and the negative‐reappraisal condition (*p* < .001). The valence of negative‐reappraisal stimuli was similar to the neutral stimuli at trend level (*p* = .062). In the failure group, the main effect of Condition (*F*(2,24) = 42.069, *p* < .001, *η* ^2^ = .778) revealed that negative‐watch stimuli elicited greater unpleasantness than the neutral stimuli (*p* < .001) and the negative‐reappraisal stimuli (*p* = .028). In addition, negative‐reappraisal stimuli elicited more unpleasantness than the neutral stimuli (*p* < .001).
2. The independent *t* tests of between‐group comparisons were performed for each condition. Negative‐reappraisal reduced more unpleasantness in the success group than the failure group (*t* = 3.915, *p* = .001), while no group effect was found for negative‐watch and the neutral stimuli.

### 3.2 ERP results

**Figure 3:**
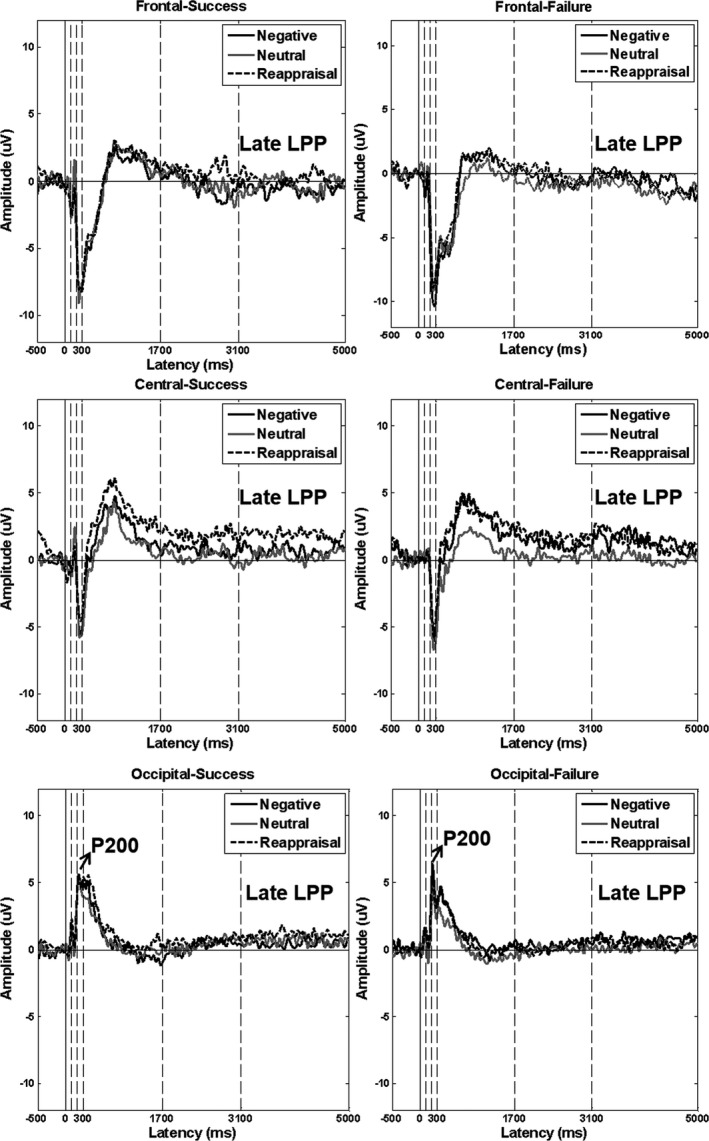
Grand average waveforms to different stimuli types recorded during the regulation task in the central region and occipital region, separately. The N100/P200 were scored in the occipital region, and LPP (300–5,000 ms after the stimulus onset) was scored in frontal, central, and occipital regions. For P200 (peak at 200–300 ms), the amplitude was increased to the negative‐watch stimuli relative to the negative‐reappraisal and the neutral stimuli in the failure group but not in the success group. For late LPP (3,100–5,000 ms), the amplitude induced by negative‐reappraisal was greater than that induced by the negative‐watch in the success group; such difference was not observed in the failure group. P200 and late LPP indexed the time windows differentiated the success and failure groups. Black solid line denotes the negative‐watch stimuli; gray solid line denotes the neutral stimuli; black dotted line denotes the negative‐reappraisal stimuli

Figure 3 presents the grand average waveforms in the frontal, central, and occipital recording sites in the success and in the failure groups, separately.

#### 3.2.1 N100 and P200

Table 3 presents the N100 and P200 amplitudes on the occipital region.

**Table 3:** N100 and P200 amplitudes of the neutral, negative‐watch and negative‐reappraisal stimuli on the occipital region for the success and failure groups

Condition	N100‐Occipital		P200‐Occipital	
	Success	Failure	Success	Failure
Neutral	1.13 (2.34)	0.44 (2.20)	6.00 (2.88)	4.95 (4.05)
Negative‐watch	1.55 (2.62)	1.25 (2.12)	6.11 (2.91)	6.88 (3.30)
Negative‐reappraisal	1.54 (2.13)	1.24 (1.86)	6.48 (3.14)	5.79 (3.71)

Note

The success and failure groups differed in P200 when they reappraised negative images.

##### N100

We found a main effect of Condition (*F*(2,46) = 3.987, *p* = .025, *η* ^2^ = .142), indicating that the N100 to the neutral stimuli was more negative than negative‐reappraisal stimuli (*p* = .032). No Group effect as well as the interaction effect of Condition*Group was found.

##### P200

We found a main effect of Condition (*F*(2,46) = 6.057, *p* = .005, *η* ^2^ = .208), indicating that the P200 to negative‐watch stimuli was more positive than the neutral stimuli (*p* = .013). Importantly, a significant interaction effect of Condition*Group (*F*(2,46) = 5.088, *p* = .010, *η* ^2^ = .181) was found. The ANOVA with a within factor of Condition was performed in each group. In the success group, no Condition effect was found (*p* = .536). In the failure group, a significant Condition effect was found (*F*(2,22) = 11.621, *p* < .001, *η* ^2^ = .514), suggesting that the P200 to negative‐watch stimuli was more positive than negative‐reappraisal stimuli (*p* = .047) and the neutral stimuli (*p* = .005). No Group effect was found.

#### 3.2.2 LPP results

Table 4 presents the LPP amplitudes in the early, middle, and late windows on the frontal, central, and occipital regions.

**Table 4:** Mean LPP amplitudes (*SD* in parentheses) of each stimulus type in the early, middle and late time windows on frontal, central and occipital regions for the success and failure groups

Group	Condition	LPP_Early			LPP_Middle			LPP_Late		
		Frontal	Central	Occipital	Frontal	Central	Occipital	Frontal	Central	Occipital
Success	Neutral	0.44 (0.92)	1.14 (0.73)	0.7 (0.45)	0.07 (0.66)	0.31 (0.51)	0.39 (0.37)	−0.2 (0.88)	0.2 (0.50)	0.67 (0.36)
	Negative‐watch	0.22 (1.03)	1.97 (0.52)	0.72 (0.61)	−0.12 (0.63)	0.76 (0.40)	0.2 (0.45)	−0.31 (0.81)	0.67 (0.38)	0.45 (0.43)
	Negative‐reappraisal	0.44 (1.13)	3.27 (0.77)	1.44 (0.44)	1.02 (0.64)	1.87 (0.52)	0.62 (0.30)	0.32 (0.97)	1.92 (0.57)	1.11 (0.31)
Failure	Neutral	−1.67 (1.10)	0.65 (0.72)	0.24 (0.40)	−0.8 (0.82)	0.27 (0.57)	−0.02 (0.26)	−1.34 (0.85)	0.1 (0.54)	0.17 (0.31)
	Negative‐watch	−1.2 (1.28)	2.37 (0.59)	1.15 (0.64)	−0.51 (0.78)	1.2 (0.46)	0.3 (0.34)	−0.39 (0.77)	1.51 (0.39)	0.7 (0.43)
	Negative‐reappraisal	−0.83 (1.14)	2.79 (0.70)	0.9 (0.51)	−0.19 (0.66)	1.76 (0.44)	0.19 (0.39)	−0.83 (0.76)	1.45 (0.48)	0.47 (0.35)

Note

The success and failure groups differed in LPP late when they reappraised negative images.

##### Early window (300–1,700 ms)

The Condition effect (*F*(2,48) = 8.683, *p* = .001, *η* ^2^ = .266) indicated that both negative‐reappraisal (*p* = .005) and negative‐watch (*p* = .074) elicited greater LPP than the neutral stimuli. The LPP to negative‐reappraisal versus negative‐watch, however, did not differ (*p* = .102). A significant Area effect (*F*(2,48) = 5.337, *p* = .013, *η* ^2^ = .182) suggested that the central LPP was significantly more positive than the frontal LPP (*p* = .002). The effect of Group (*p* = .875) and the interaction effect of Condition*Group (*p* = .126) did not reach significance.

A significant interaction effect of Condition*Area (*F*(4,96) = 4.608, *p* = .003, *η* ^2^ = .161) was found. To follow up on the Condition*Area interaction, the ANOVA with a within factor of condition was performed in each area. This analysis suggested significant Condition effect in the central region (*F*(2,50) = 17.379, *p* < .001, *η* ^2^ = .410) and in the occipital region (*F*(2,50) = 5.503, *p* = .011, *η* ^2^ = .180), respectively. In the central region, the LPP to negative‐reappraisal was increased compared with negative‐watch (*p* = .037), and the LPP to both negative‐watch (*p* = .004) and negative‐reappraisal (*p* < .001) was more positive than to the neutral stimuli. In the occipital region, the LPP to negative‐reappraisal was more positive than to the neutral stimuli (*p* = .001). There was no significant finding in the frontal region.

##### Middle window (1,700–3,100 ms)

A significant Condition effect (*F*(2,48) = 6.249, *p* = .004, *η* ^2^ = .207) was found. The LPP to negative‐reappraisal was more positive than in the negative‐watch condition (*p* = .052), and to the neutral stimuli (*p* = .013). The difference between negative‐watch and the neutral stimuli was not significant (*p* = .810). An Area effect at a trend level (*F*(2,48) = 2.972, *p* = .075, *η* ^2^ = .110) was also found. The effect of Group (*p* = .923) and the interaction effect of Condition*Group (*p* = .211) did not reach significance.

There was an interaction effect of Condition*Area (*F*(4,96) = 3.426, *p* = .016, *η* ^2^ = .125). To follow up on the Condition*Area interaction, the ANOVA with a within factor of condition was performed for each area. A significant Condition effect (*F*(2,50) = 11.390, *p* < .001, *η* ^2^ = .313) was only found in the central region, where the LPP to negative‐reappraisal was more positive than to negative‐watch (*p* = .029) and to the neutral stimuli (*p* = .001).

##### Late window (3,100–5,000 ms)

The main effect of Condition (*F*(2,48) = 6.455, *p* = .003, *η* ^2^ = .212) suggested that the LPP to negative‐reappraisal was more positive than to the neutral stimuli (*p* = .015). The main effect of Area (*F*(2,48) = 3.770, *p* = .042, *η* ^2^ = .136) suggested that the central LPP was greater than the frontal LPP (*p *= .011). The effect of Group did not reach significance (*p* = .352).

Importantly, we found significant interaction effects of Condition* Group (*F*(2,48) = 3.333, *p* = .044, *η* ^2^ = .122) as well as Condition*Area (*F*(4,96) = 2.685, *p* = .036, *η* ^2^ = .101).

To follow up on the interaction effect of Condition*Group, we conducted further analyses:

1. The ANOVA with a within factor of Condition was performed in each group. In the success group, a significant Condition effect (*F*(2,24) = 5.656, *p* = .010, *η* ^2^ = .320) showed that the LPP to the negative‐reappraisal stimuli was more positive than to the negative‐watch (*p* = .020) and to the neutral stimuli (*p* = .072) at trend level. In the failure group, a Condition effect (*F*(2,24) = 4.311, *p* = .025, *η* ^2^ = .264) showed that the LPP to negative‐watch stimuli was more positive at trend level than to the neutral stimuli (*p* = .071), while no difference was found between negative‐watch and negative‐reappraisal stimuli.
2. The independent *t* tests in between‐group comparisons were performed for each condition. No between‐group findings were shown in each condition (*p* > .05).

To follow up on the interaction effect of Condition*Area, the ANOVA with a within factor of Condition was performed in each area. The results suggested a significant Condition effect (*F*(2,50) = 12.436, *p* < .001, *η* ^2^ = .332) only in the central region. The follow‐up analyses indicated that the LPP to the negative‐reappraisal (*p* < .001) and the negative‐watch stimuli (*p* = .017) was more positive than to the neutral stimuli, while no difference was found between negative‐reappraisal and negative‐watch.

### 3.3 Correlations

For the LPP average amplitude, in each of the latency windows, we did not find any correlations with the valence/arousal difference between the negative‐watch and the negative‐reappraisal conditions in either the success group or the failure group.

## 4 DISCUSSION

The results of this study suggest that reappraisal success has a different neural signature than reappraisal failure. In the success group, the LPP amplitude indexed the difference between negative‐watch and negative‐reappraisal starting from 300 ms to the end of the epoch. In the failure group, the difference between negative‐watch and negative‐reappraisal was associated with the P200, while the LPP amplitude indexed the differences between the negative‐watch and negative‐reappraisal conditions starting from 300 to 3,100 ms, and thus was similar to the success group, but such difference was lacking in the late epoch (3,100–5,000 ms).

### 4.1 Behavioral effects

In the current study, both reappraisal success and failure groups processed the negative‐watch stimuli as equally unpleasant, and they had similar arousal ratings, consistent with previous research (Foti & Hajcak, 2008; Hajcak & Nieuwenhuis, 2006; Thiruchselvam et al., 2011; Van Cauwenberge et al., 2017; Yuan et al., 2014). These results suggest that both groups were equally impacted by the negative stimuli.

However, the two groups showed a differential behavioral response in the negative‐reappraisal condition: The success group reduced the level of unpleasantness and arousal associated with the negative stimuli more than the failure group. Our results demonstrated that use of the strategy of cognitive reappraisal modulated self‐reported ratings of valence and arousal of the negative stimuli.

In contrast, while the failure group could rate the negative‐reappraisal stimuli as less unpleasant than the negative‐watch stimuli, they could not reduce the arousal of negative‐reappraisal stimuli relative to the negative‐watch stimuli. Previous studies (Gardener et al., 2013; Sarlo et al., 2013) that reported on reappraisal failures also found that unsuccessful reappraisal was associated with the failure to reduce the arousal of negative images based on self‐report.

Our behavioral results provided a direct evidence that some individuals can deploy the reappraisal strategy successfully but others cannot.

### 4.2 P200 effects

In the present study, the failure group showed a significantly larger P200 to negative‐watch relative to negative‐reappraisal and neutral stimuli. These P200 effects were entirely absent in the success group. P200 is associated with early attention processes indexing automatic capture of attention toward affective stimuli (Huang & Luo, 2006). In addition, P200 is associated with early categorization processes where upcoming stimuli are tagged for their emotional valence but without overt categorization into types of emotional experience (Olofsson, Nordin, Sequeira, & Polich, 2008). These results suggest that in the failure group, negative images in the watch condition captured the attention more than the negative stimuli in the reappraisal and neutral conditions. Since both sets of negative images were equated for valance and arousal, it is unlikely that this effect was due to the properties of the images. It is possible that the two groups differed on the dimensions which were not captured by the demographic and clinical measures used in the study. We can also speculate that, in the failure group, the reappraisal strategy used in the reappraisal condition impacted early categorization processes, as indexed by the P200 reduced to the negative‐reappraisal condition relative to the negative‐watch condition. In contrast, in the success group, no condition differences were observed for the P200 suggesting that the early processes did not differentiate between the different strategies deployed to watch and reappraise the images.

### 4.3 Increased LPPs to reappraisal of negative stimuli

Consistent with previous research (DeCicco, Solomon, & Dennis, 2012; Thiruchselvam et al., 2011), the negative images induced greater LPPs relative to neutral images. This finding was localized to the central region. These results support the finding that LPP is sensitive to emotional stimuli and typically maximal at central–parietal sites (Hajcak & Nieuwenhuis, 2006; Schupp et al., 2000, 2003).

In the current study, in addition to the group differences in the P200 described above, reappraisal of negative stimuli was associated with increased LPP amplitude relative to both negative‐watch and neutral stimuli in both subject groups. A number of previous studies reported that reappraisal of negative stimuli reduced the amplitude of LPP relative to the passive viewing of negative stimuli (Foti & Hajcak, 2008; Hajcak & Nieuwenhuis, 2006; Parvaz et al., 2012; Thiruchselvam et al., 2011). Nevertheless, a few recent studies failed to demonstrate this effect of reappraisal on the LPP amplitude in both normal adults (Gardener et al., 2013; Sarlo et al., 2013; Yuan et al., 2014) and young children (DeCicco, O'Toole, & Dennis, 2014; DeCicco et al., 2012; Van Cauwenberge et al., 2017).

There are important differences between the methodology used in our study and in those studies (Langeslag & Surti, 2017) that found LPP enhancement related to reappraisal strategies, relative to the studies that found the LPP reduction (Foti & Hajcak, 2008; Hajcak & Nieuwenhuis, 2006; Moser, Hartwig, Moran, Jendrusina, & Kross, 2014; Parvaz et al., 2012; Thiruchselvam et al., 2011). In the studies that found the LPP reduction (Hajcak & Nieuwenhuis, 2006; Moser et al., 2014; Parvaz et al., 2012), the study design involved the repetition of the same negative stimuli that were presented in the negative‐watch condition under the reappraisal watch conditions. While the authors made an argument that the repetition did not contribute to the LPP reduction, it seems unlikely that it did not contribute to the LPP effect. In other studies (Foti & Hajcak, 2008; Macnamara et al., 2009), the participants were asked to engage in reappraisal strategies before they were exposed to the negative images. These approaches result in the actual reappraisal processes preceding the exposure to the negative stimuli by changing the emotional “tone” or emotional states.

In contrast, our study, as well as Langeslag and Surti study (2017), that found the LPP enhancement used the strategies that capture the reappraisal processes as they happen in real time by asking the participants to engage in the reappraisal strategies at the onset of the negative stimuli. LPP indexes effortful attention and reprocessing, assigning the ultimate meaning, including emotional meaning. The studies that found the LPP reduction demonstrated that less effort is needed to reinterpret negative images if the neurocognitive strategies aimed to change an emotional reaction start before the onset of a negative image. Our study demonstrated that the cognitive processes that facilitate a less negative reaction take more effort than those involved in just watching the negative images or neutral ones. The two types of studies offer important insights into the dynamics of affective reappraisal, with each providing a different temporal window into the interactions between cognitive and affective systems in achieving adoptive emotional states such as reduction of negative emotion. As the study of Moser et al. (2009) argues, the exact cognitive conditions, or context, within which reappraisal happens matter: These variables impact the temporal unfolding of neural and cognitive events leading to successful, or unsuccessful, reappraisal.

### 4.4 Reappraisal failure failed to maintain the reappraisal of negative stimuli in the late time

The LPP amplitude changes between the negative‐watch and the negative‐reappraisal condition were found starting 300 ms after the stimulus onset in the central region, in both reappraisal success and failure groups. According to the Kalisch's implementation–maintenance model (2009), reappraisal involves different stages: The early stage includes the implementation of an initial reappraisal strategy, while the late stage includes the operations needed to maintain that strategy and to monitor its success during the course of an emotional situation.

The early latency window LPP results suggest that both the success and failure groups implemented an initial reappraisal strategy. The success group maintained that strategy for the rest of the recording epoch. However, in the failure group, the lack of LPP amplitude differences between the negative‐watch and negative‐reappraisal stimuli suggests that this group failed to maintain the reappraisal strategy to the end of the recording epoch, that is, over the late LPP latency window (3,100–5,000 ms). As a consequence, this group was not successful in decreasing the impact of negative images.

These results provide electrophysiological evidence that, as suggested by the Kalisch model, reappraisal success is predicated on the implementation of reappraisal strategies and a continuous effort to adjust the reappraisal in the course of maintenance/monitoring (late) operations, which was reflected in the increased LPP amplitudes in the current study. This temporally extended and dynamic process would lead to a successful reduction in the quality (valence) and quantity (arousal) of a negative response to negative images. Thus, this study, like others, suggests that it is possible to manage/modulate negative reactions to negative emotions and that deploying appropriate cognitive strategies serves this purpose.

As discussed in the Introduction, to the best of our knowledge, this is the first study to examine ERP correlates of both success and failure of negative images reappraisal. Therefore, the current results cannot be easily compared to those reported in the existing published studies.

### 4.5 No correlation was found between the LPP and the behavioral ratings

Some previous studies reported a correlation between neural activity and phenomenological experience in terms of cognitive reappraisal. For instance, Hajcak and Nieuwenhuis (2006) suggested that the reduction in LPP amplitude was positively correlated with a reduction in the self‐reported emotional intensity. Ray et al. (2010) also found a positive correlation between self‐reports of affective experience and startle EMG measures in the down‐regulation reappraisal of the negative stimuli. However, several studies failed to find such a link (Foti & Hajcak, 2008; Thiruchselvam et al., 2011). In the present study, we did not find a correlation between the LPP and self‐reported emotional intensity. Further investigations are necessary to explore the link between neural correlates and behavioral ratings in cognitive reappraisal.

### 4.6 Limitations of the study

In the current experimental paradigm, the participants reappraised the negative images using both the detachment (imagine the negative image to be in the movie) and positive re‐interpretation (imagine a positive outcome). Thus, it is not clear exactly which strategy the participants used to mitigate negative emotions toward negative images and whether these strategies differed between the success and failure groups. Future experiments should assign participants to the two categories of reappraisal of negative stimuli to arrive at more definitive answers.

In future research, we will consider assigning participants to a specific strategy in re‐evaluating the content of an emotional stimulus. In addition, cognitive reappraisal should be used not only for negative stimuli but also for neutral and positive stimuli in order to fully understand the neural mechanisms of cognitive reappraisal. Finally, a larger number of participants will contribute to more robust results and help verity findings of the present study.

## 5 CONCLUSIONS

To the best of our knowledge, this is the first study to investigate the neutral characteristics of reappraisal success and failure in one group of individuals, at the same time. Our study indicated that the success and failure groups showed different electrophysiological characteristics while engaging in the reappraisal of negative stimuli. Although both reappraisal success and failure groups showed the same LPP amplitude changes from 300 to 3,100 ms after image onset, cognitive reappraisal success was associated with the increased LPP relative to negative‐watch beginning at 3,100 ms; such an increase was not observed in the failure group. These results suggest that different neural activities result in different behavioral outcomes, even if direct correlations between the neural and behavioral levels are not present.

## CONFLICT OF INTEREST

None of the authors have potential conflicts of interest to be disclosed.

## AUTHOR CONTRIBUTION

Y.L. developed the study concept. D.C. developed the study design and performed data collection and analysis. M.A.N. made significant contributions to data interpretation. All authors contributed to manuscript writing and revision. All authors approved the final version of the manuscript for submission.

## Data Availability

The data that support the findings of this study are available from the corresponding author upon reasonable request.
